# Supplementary material for: The Evolution and Characterization of the RNA Interference Pathways in Lophotrochozoa
Source: Genome Biol Evol. 2024 May 7;16(5):evae098. doi: 10.1093/gbe/evae098 (PMC11114477; doi:10.1093/gbe/evae098)
Supplement: evae098_Supplementary_Data [file evae098_supplementary_data.zip › Supplementary_materials.zip!/selected_miRNAs/NoGe_selected_miRNAs/mirdeep_pdf_files/NMRB01000201.1_Notospermus_geniculatus_strain_Ushimado_scaffold201,_whole_genome_shotgun_sequence_7104.pdf]

Provisional ID : NMRB01000201.1_Notospermus_geniculatus_strain_Ushimado_scaffold201,_whole_genome_shotgun_sequence_7104

Score total	:	9774.9
Score for star read(s)	:	3.9
Score for read counts	:	9766.3
Score for mfe	:	1.7
Score for randfold	:	0
Score for cons. seed	:	3
Total read count	:	19168
Mature read count	:	19081
Loop read count	:	0
Star read count	:	87

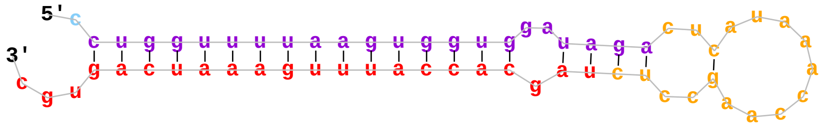

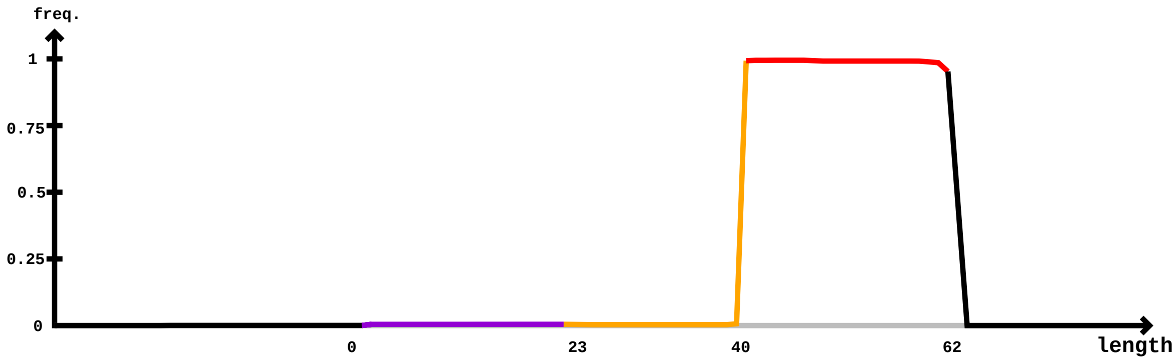

Star Mature

```
5'- gaugaugccacagaauauggacccgaggagccugguuuuaagugguggauagacucauaaaccaagccucuagcaccauuugaaaucagugcugcugggcguugcuagucga  -3'  obs
    gaugaugccacagaauauggacccgaggagccugguuuuaagugguggauagacucauaaaccaagccucuagcaccauuugaaaucagugcugcugggcguugcuagucga       exp
    ............((.((..(((((.((.(((((((((((((((((((..((((..(.........)..)))).)))))))))))))))).))).)).)).)))..)).))..  reads  mm  sample
    ..........cagaauauggacccgaggagc.................................................................................  4      0   Mm2
    ...............................cugguuuuaagugguggauaga...........................................................  15     0   Mm2
    ...............................cugguuuuaagugguggauagacucauaaaccaagccu...........................................  1      0   Mm2
    ..........................................ugguggauagacucauaaaccaagccu...........................................  2      0   Mm2
    ..............................................ggauagacucauaaaccaagccu...........................................  1      0   Mm2
    .....................................................cucauaaaccaagccucuGgcacc...................................  1      1   Mm2
    .....................................................cucauaaaccaagccucuGgcacca..................................  1      1   Mm2
    ......................................................ucauaaaccaagccucuGgcacca..................................  1      1   Mm2
    .....................................................................cuGgcaccauuugaaaucagug.....................  3      1   Mm2
    .....................................................................cuGgcaccauuugaaaucagugc....................  1      1   Mm2
    ......................................................................uagcaccauuugaaaucagu......................  1      0   Mm2
    ......................................................................uGgcaccauuugaaaucagu......................  3      1   Mm2
    ......................................................................uCgcaccauuugaaaucagug.....................  1      1   Mm2
    ......................................................................uagcaccauuugaaaucagug.....................  19     0   Mm2
    ......................................................................uGgcaccauuugaaaucagug.....................  46     1   Mm2
    ......................................................................uagcaccauuugaaaucaAugc....................  1      1   Mm2
    ......................................................................uGgcaccauuugaaaucagugc....................  1118   1   Mm2
    ......................................................................uagcaccauuugaaaucagugc....................  450    0   Mm2
    ......................................................................uagcaccauuugaaaucagCgc....................  1      1   Mm2
    ......................................................................uagcaccauuAgaaaucagugc....................  2      1   Mm2
    ......................................................................uagcaccauuugaaauAagugc....................  4      1   Mm2
    ......................................................................uagcaccauuugaaaucCgugc....................  1      1   Mm2
    ......................................................................uagcaccauuugaaauUagugc....................  2      1   Mm2
    ......................................................................uCgcaccauuugaaaucagugc....................  1      1   Mm2
    ......................................................................uagcaccauuugaaauGagugc....................  2      1   Mm2
    ......................................................................uagcaccauuugaaaucagugG....................  7      1   Mm2
    ......................................................................uagcaccauuugaaaucaguAc....................  1      1   Mm2
    ......................................................................uagcaccauuugaaaucUgugc....................  1      1   Mm2
    ......................................................................Nagcaccauuugaaaucagugc....................  1      1   Mm2
    ......................................................................uagcGccauuugaaaucagugc....................  5      1   Mm2
    ......................................................................uagcaccauuugaaaucGgugc....................  3      1   Mm2
    ......................................................................uGgcaccauuugaaaucagugcu...................  3      1   Mm2
    ......................................................................uagcaccauuugaaaucagugcA...................  1      1   Mm2
```

Star **Mature**

gaugaugccacagaauauggacccgaggagccugguuuuaagugguggauagacucauaaaccaagccucuagcaccauuugaaaucagugcugcugggcguugcuagucga			
....uagcaccauuugaaaucaguUcu...................	17	1	Mm2
....Ggcaccauuugaaaucagugc....................	1	1	Mm2
..........cagaauauggacccgaggagc...	2	0	Mm1
....uagcaccauuugaaaucagu......................	3	0	Mm1
....uagcaccauuugaaaucUgug.....................	1	1	Mm1
....uagcaccauuugaaaucagug.....................	137	0	Mm1
....uagcaccauuugaaauAagug.....................	1	1	Mm1
....uGgcaccauuugaaaucagugc....................	88	1	Mm1
....uagcaccauuugaaaucCgugc....................	1	1	Mm1
....uagcaccauuugaaaucagugc....................	24	0	Mm1
....uagcaccauuugaaaucaguUcu...................	3	1	Mm1
....uGgcaccauuugaaaucagugcu...................	1	1	Mm1
....uagcaccauuugaaaucagugcA...................	10	1	Mm1
..........cagaauauggacccgaggagc...	1	0	Fm1
...............................cugguuuuaagugguggauaga...	39	0	Fm1
...............................cugguuuuaagugguggauagU...	1	1	Fm1
....ucauaaaccaagccucuGgcacca..................................	10	1	Fm1
....cuGgcaccauuugaaaucagugc....................	9	1	Fm1
....uagcaccauuugaaauca........................	4	0	Fm1
....uGgcaccauuugaaaucagug.....................	32	1	Fm1
....uagcaccauuugaaaucagug.....................	4	0	Fm1
....uagcaccauuugaaaucagugG....................	43	1	Fm1
....uagcaccauuugaaaucagugA....................	2	1	Fm1
....uUgcaccauuugaaaucagugc....................	3	1	Fm1
....uagcaccauuugaaaucaUugc....................	1	1	Fm1
....uagcaccauuugaCaucagugc....................	1	1	Fm1
....uagcaccauuugaaaucGgugc....................	10	1	Fm1
....uagcaccauuugaaauGagugc....................	2	1	Fm1
....uagcaccauuuGGaaucagugc....................	1	1	Fm1
....uagcaccUuuugaaaucagugc....................	1	1	Fm1
....uagcaccauuugaaaucUgugc....................	13	1	Fm1
....uagcaccauuGgaaaucagugc....................	4	1	Fm1
....uagcaccauuugaaauAagugc....................	20	1	Fm1
....uagcacAauuugaaaucagugc....................	1	1	Fm1
....uagcaccauuugaaauUagugc....................	4	1	Fm1
....uaUcaccauuugaaaucagugc....................	2	1	Fm1
....uagcaccauuAgaaaucagugc....................	6	1	Fm1
....uagcaccauuugaGaucagugc....................	2	1	Fm1
....uagcaccauuCgaaaucagugc....................	2	1	Fm1
....uagcacUauuugaaaucagugc....................	2	1	Fm1
....uagcaccauuugaaaucagugc....................	3148	0	Fm1
....uagcGccauuugaaaucagugc....................	6	1	Fm1
....uagcaGcauuugaaaucagugc....................	1	1	Fm1
....uagcaccauuugaaaucagugU....................	2	1	Fm1
....uGgcaccauuugaaaucagugc....................	9688	1	Fm1
....uagcaUcauuugaaaucagugc....................	2	1	Fm1
....uagcaccauuugaaaucCgugc....................	15	1	Fm1
....Nagcaccauuugaaaucagugc....................	1	1	Fm1
....uagcaccauuugaaaucaguUcu...................	1	1	Fm1
....uGgcaccauuugaaaucagugcu...................	1	1	Fm1
....uagcaccauuugaaaucagugcA...................	3	1	Fm1
....uagcaccauuugaaaucagugcu...................	11	0	Fm1
....Ggcaccauuugaaaucagugc....................	13	1	Fm1
....agcaccauuugaaaucagugc....................	8	0	Fm1
....ucauaaaccaagccucuGgcacc...................................	29	1	Fm2
....ucauaaaccaagccucuGgcacca..................................	16	1	Fm2
....cuGgcaccauuugaaaucagug.....................	19	1	Fm2
....cuGgcaccauuugaaaucagugc....................	19	1	Fm2
....uGgcaccauuugaaaucagug.....................	13	1	Fm2
....uagcaccauuugaaauGagug.....................	1	1	Fm2
....uagcaccauuugaaaucagug.....................	14	0	Fm2
....uagcaccauuugaaaucagugA....................	1	1	Fm2
....uagcaccauuugaaaucagugG....................	4	1	Fm2
....uagcaccauuugaaauUagugc....................	1	1	Fm2
....uGgcaccauuugaaaucagugc....................	726	1	Fm2
....uagcaccauuugaaauAagugc....................	3	1	Fm2
....uCgcaccauuugaaaucagugc....................	2	1	Fm2

Star **Mature**

gaugaugccacagaauauggacccgaggagccugguuuuaagugguggauagacucauaaaccaagcccucuagcaccauuugaaaucagugcugcugggcguugcuagucga			
....uUgcaccauuugaaaucagugc....................	1	1	Fm2
....Aagcaccauuugaaaucagugc....................	1	1	Fm2
....uagcaccauuugaaaucagugc....................	248	0	Fm2
....uagcaccauuugaaaucCgugc....................	1	1	Fm2
....uagcaccauuugaaaucagugcA...................	10	1	Fm2
....uagcaccauuugaaaucaguUcu...................	9	1	Fm2
...............................cugguuuuaagugguggauaga..	8	0	Mm3
....ugguggauagacucauaaaccaagcccu...	1	0	Mm3
....ucuGgcaccauuugaaaucagug.....................	1	1	Mm3
....cuGgcaccauuugaaaucagug.....................	5	1	Mm3
....cuGgcaccauuugaaaucagugc....................	1	1	Mm3
....cuagcaccauuugaaaucagugc....................	1	0	Mm3
....uagcaccauuugaaaucagu......................	16	0	Mm3
....uGgcaccauuugaaaucagu......................	7	1	Mm3
....uagcaccauuugaaaucagug.....................	20	0	Mm3
....uagcaccauuugaaauUagug.....................	1	1	Mm3
....uGgcaccauuugaaaucagug.....................	25	1	Mm3
....uGgcaccauuugaaaucagugc....................	334	1	Mm3
....uUgcaccauuugaaaucagugc....................	1	1	Mm3
....Cagcaccauuugaaaucagugc....................	1	1	Mm3
....uagcaccauuAgaaaucagugc....................	1	1	Mm3
....uagcaccauuGgaaaucagugc....................	1	1	Mm3
....uagcaccauuugaaaucagugc....................	204	0	Mm3
....uagcaccauuugaaauGagugc....................	1	1	Mm3
....uagcaccauuugaaaucGgugc....................	1	1	Mm3
....uagcaccauuugaaaucaguUcu...................	28	1	Mm3
....uagcaccauuugaaaucagugcA...................	2	1	Mm3
....agcaccauuugaaaucagug.....................	1	0	Mm3
....caccauuugaaaucagugc....................	1	0	Mm3
...............................cugguuuuaagugguggauag...	3	0	Fm3
...............................cugguuuuaagugguggauaga..	21	0	Fm3
....ucuGgcaccauuugaaaucagug.....................	2	1	Fm3
....cuGgcaccauuugaaaucagug.....................	8	1	Fm3
....cuGgcaccauuugaaaucagugc....................	4	1	Fm3
....uagcaccauuugaaaucaU.......................	1	1	Fm3
....uagcaccauuugaaaucag.......................	52	0	Fm3
....uaUcaccauuugaaaucagu......................	4	1	Fm3
....uagcaccauuuAaaaucagu......................	1	1	Fm3
....uagcaccauuugaaaucagu......................	27	0	Fm3
....uGgcaccauuugaaaucagu......................	1	1	Fm3
....uagcaccauuugaGaucagug.....................	1	1	Fm3
....uagcaccauuugaaaucCgug.....................	1	1	Fm3
....uagcaccauuAgaaaucagug.....................	2	1	Fm3
....uagcaccauuugaaaucaguA.....................	1	1	Fm3
....uagcaccauuugaaaucagCg.....................	1	1	Fm3
....uagcaccauuugaaaucGgug.....................	3	1	Fm3
....uagcaccauuugaaauUagug.....................	2	1	Fm3
....uagcaccauuugaaauAagug.....................	2	1	Fm3
....uagcaccauuugaaaucagug.....................	210	0	Fm3
....uGgcaccauuugaaaucagug.....................	55	1	Fm3
....uagcaccauuugaaaucagugA....................	3	1	Fm3
....uagcaccauuugaaaucaAugc....................	1	1	Fm3
....uagcaccauuugaaauGagugc....................	2	1	Fm3
....Cagcaccauuugaaaucagugc....................	1	1	Fm3
....uagcaccauuugaaauAagugc....................	4	1	Fm3
....uagcaccauuCgaaaucagugc....................	3	1	Fm3
....uagcaccauuugaaaucaguAc....................	1	1	Fm3
....uagcaccauuugaaauUagugc....................	1	1	Fm3
....uagcaccauuugaaaucCgugc....................	3	1	Fm3
....uagcaccauuGgaaaucagugc....................	2	1	Fm3
....uagcaccauuugaaaucGgugc....................	1	1	Fm3
....uagcaccauuugaaaucagugc....................	587	0	Fm3
....uagcGccauuugaaaucagugc....................	1	1	Fm3
....uagcaccauuAgaaaucagugc....................	1	1	Fm3
....uGgcaccauuugaaaucagugc....................	1300	1	Fm3
....uagcaccauuugaaaucUgugc....................	3	1	Fm3
....uagcaccauuugaaaucagugU....................	7	1	Fm3
....uGgcaccauuugaaaucagugcu...................	3	1	Fm3

Star Mature

gaugaugccacagaauauggacccgaggagccugguuuuaagugguggauagacucauaaaccaagccucuagcaccauuugaaaucagugcugcugggcguugcuagucga			
....uagcaccauuugaaaucaguUcu...................	8	1	Fm3
....uagcaccauuugaaaucagugcu...................	2	0	Fm3
....agcaccauuugaaaucagug.....................	1	0	Fm3
....agcaccauuugaaaucagugc....................	2	0	Fm3
....Ggcaccauuugaaaucagugc....................	3	1	Fm3
....caccauuugaaaucagugcu...................	3	0	Fm3
